# Polygenic scores for handedness and their association with asymmetries in brain structure

**DOI:** 10.1007/s00429-021-02335-3

**Published:** 2021-07-08

**Authors:** Sebastian Ocklenburg, Dorothea Metzen, Caroline Schlüter, Christoph Fraenz, Larissa Arning, Fabian Streit, Onur Güntürkün, Robert Kumsta, Erhan Genç

**Affiliations:** 1 Department of Biopsychology, Faculty of Psychology, Institute of Cognitive Neuroscience, Ruhr University Bochum, Bochum, Germany; 2 Leibniz Research Centre for Working Environment and Human Factors (IfADo), Department of Psychology and Neurosciences, Dortmund, Germany; 3 Department of Human Genetics, Faculty of Medicine, Ruhr University Bochum, Bochum, Germany; 4 Medical Faculty Mannheim, Department of Genetic Epidemiology in Psychiatry, Central Institute of Mental Health, University of Heidelberg, Mannheim, Germany; 5 Department of Genetic Psychology, Faculty of Psychology, Ruhr University Bochum, Bochum, Germany

**Keywords:** Handedness, Laterality, Hemispheric asymmetry, Polygenic scores, Genetics, Motor

## Abstract

Handedness is the most widely investigated motor preference in humans. The genetics of handedness and especially the link between genetic variation, brain structure, and right-left preference have not been investigated in detail. Recently, several well-powered genome-wide association studies (GWAS) on handedness have been published, significantly advancing the understanding of the genetic determinants of left and right-handedness. In the present study, we estimated polygenic scores (PGS) of handedness-based on the GWAS by de Kovel and Francks (Sci Rep 9: 5986, 2019) in an independent validation cohort (*n* = 296). PGS reflect the sum effect of trait-associated alleles across many genetic loci. For the first time, we could show that these GWAS-based PGS are significantly associated with individual handedness lateralization quotients in an independent validation cohort. Additionally, we investigated whether handedness-derived polygenic scores are associated with asymmetries in gray matter macrostructure across the whole brain determined using magnetic resonance imaging. None of these associations reached significance after correction for multiple comparisons. Our results implicate that PGS obtained from large-scale handedness GWAS are significantly associated with individual handedness in smaller validation samples with more detailed phenotypic assessment.

**Supplementary Information:**

The online version contains supplementary material available at 10.1007/s00429-021-02335-3.

## Introduction

Overall, 10.6% of the general population are left-handers (Papadatou–Pastou et al. 2020), making the distribution of upper limb preferences in humans decidedly more right-skewed than those in almost all non-human animal species (Ströckens et al. 2013). Despite more than a century of research on left-handedness, the ontogenesis of handedness still is not well understood (Ocklenburg et al. 2013). Regardless of earlier attempts to characterize handedness as a monogenic phenotype (Annett 1996; McManus 1991), it is now generally accepted that handedness is a complex phenotype that is determined by a multitude of possibly interacting, genetic and non-genetic factors (Güntürkün and Ocklenburg 2017; de Kovel et al. 2019; Ocklenburg et al. 2013; Paracchini et al. 2016; Schmitz et al. 2017). Twin studies suggest that about a quarter of the variance in handedness data can be explained by additive genetics factors (Medland et al. 2006, 2009).

Over the years, candidate gene studies have associated several genes with hand preference and hand skill, e.g., *LRRTM1* (Francks et al. 2007; Leach et al. 2014), *PCSK6* (Arning et al. 2013; Brandler et al. 2013; Scerri et al. 2011), and *SETDB2* (Crespi et al. 2018; Ocklenburg et al. 2016), among others. However, these associations could not be replicated in newer genome-wide association studies (GWAS) applying genome-wide significance thresholds corrected for multiple comparisons (Cuellar–Partida et al. 2020; de Kovel and Francks 2019; Wiberg et al. 2019). This pattern of results does not come entirely unexpected, as the results of candidate gene studies of complex behavioral traits often fail to replicate in independent cohorts (Knopik et al. 2017). Instead, each of these three large-scale genome-wide association studies on handedness utilizing the UK Biobank and other datasets identified novel significant associations with handedness. The first of the three GWAS (de Kovel and Francks 2019) used the UK Biobank cohort, with an overall *N* of 331,037 included in the analysis. In this study, the authors conducted three different GWAS: left-handed vs non-left-handed, right-handed vs non-right-handed and ambidextrous vs non-ambidextrous. The left-handed vs non-left-handed GWAS identified three novel loci associated with left-handedness that were located on 2q34 (lead SNP rs142367408), 17q21 (lead SNP rs144216645), and 13q22 (lead SNP rs11454570). The causative gene for the 17q21 location could not be identified as the region spanned at least twelve genes. The locus on 13q22 was annotated with *LINC00381*, a non-coding RNA-gene of unknown function. For the 2q34 location, the most proximate gene was *MAP2*, a gene associated with neurogenesis (Harada et al. 2002). The right-handed vs non-right-handed GWAS identified the same loci as the left-handed vs non-left-handed GWAS, while the ambidextrous vs non-ambidextrous GWAS did not yield any genome-wide significant results.

The second of the three GWAS (Wiberg et al. 2019) also utilized the UK Biobank dataset, but had a slightly larger sample size. Comparable to the paper by de Kovel and Francks (2019), a left-handed vs non-left-handed GWAS and right-handed vs non-right-handed GWAS were conducted. However, instead of ambidextrous vs non-ambidextrous GWAS, a right-handed vs left-handed GWAS excluding ambidextrous individuals was conducted as third analysis. Both the left-handed vs. non-left-handed GWAS and the left-handed vs. right-handed GWAS yielded three significant loci located on 17q21 (SNP rs199512), 22q11 (SNP rs45608532), and 2q34 (SNP rs13017199). The right-handers vs non-right-handers GWAS replicated that association of rs199512 and identified a further locus on 6p21 (SNP rs3094128). The rs13017199 variant is an expression quantitative trait locus of *MAP2*, a gene that was also associated with a significant locus in the study by de Kovel and Francks (2019). The last and most recent of the three GWAS (Cuellar–Partida et al. 2020) used data from the UK Biobank that were also included in the two previous GWAS, but also additional datasets from 23andMe and the International Handedness Consortium, resulting in a sample size of *N* = 1,766,671. In this study, 41 different loci were associated with left-handedness and seven loci were specifically associated with ambidexterity. Comparable to the first two handedness GWAS, one of the top hits was associated with the gene *MAP2*.

Taken together, the three GWAS imply that the results of earlier candidate gene studies on handedness likely were false positives as none of them replicated in any of the three studies. In the study by de Kovel and Francks (2019) none of the variants described above reached genome-wide or even nominal significance. This finding questions the validity of using a candidate gene approach in smaller neurogenetic studies on handedness and hemispheric asymmetries in general. However, it could still be highly important to include measures of genetic variability in empirical studies on handedness and hemispheric asymmetries, even if their cohort size is several degrees smaller than the studies described above. This is particularly true for neurogenetic studies with a deep phenotyping approach. Many large-scale datasets like the UK Biobank only contain simple handedness phenotyping, for example writing hand assessment instead of a more detailed handedness assessment with several items like the Edinburgh Handedness Inventory (EHI) (Oldfield 1971). Moreover, whereas a substantial amount of neuroimaging data have been collected in a subset of participants in the UK Biobank, more specialized fMRI paradigms for the assessment of functional hemispheric asymmetries such as fMRI dichotic listening tasks (Kompus et al. 2012) are lacking. Since such paradigms are often time-consuming and interest in them is largely limited to the laterality research community, it is unlikely that there will be large-scale datasets with several 10,000 s or 100,000 s of participants with such phenotypes in the foreseeable future. Therefore, assessing alternatives to the candidate gene approach to include measures of individual genetic variability in smaller-scale studies is an important step for laterality research.

One promising approach is to use so-called polygenic scores (PGS). PGS are scores that reflect the sum effect of trait-associated alleles across many genetic loci for each individual in a target sample (Wray et al. 2014). Importantly, PGS are informed by the results of a discovery GWAS as the weight of each loci included in the PGS is determined by the effect sizes estimated in the GWAS. PGS have been successfully used both in clinical research (Agerbo et al. 2021; Agnew–Blais et al. 2021) and cognitive neuroscience studies in healthy subjects (Engen et al. 2020; Lee et al. 2018). As PGS are based on well-powered GWAS and can be applied robustly in small samples (Dima and Breen 2015), they avoid generating spurious, non-replicable results, a common problem in candidate gene studies.

The aim of the present study was to test whether PGS based on the summary statistics of the GWAS by de Kovel and Francks (2019) are significantly associated with individual handedness lateralization quotients in an independent validation cohort. PGS for left-handedness, right-handedness, and ambilaterality were constructed as the weighted sums of each participant’s trait-associated alleles across the whole genome based on the three GWAS described in the study by de Kovel and Francks (2019). In addition, we also assessed the associations of these handedness-based PGS with structural asymmetries in gray matter volume, thickness, and surface area across the whole brain. It has been suggested that functional hemispheric asymmetries such as handedness have their physiological origin in brain structure, e.g., differences in gray matter structure (Amunts et al. 2000) or callosal connectivity (Karolis et al. 2019). Therefore, assessing the association of handedness PGS and asymmetries in gray matter structure could be informative for understanding the biological pathways in which genetic variation reflected by the PGS ultimately affects a complex behavioral phenotype like handedness. For handedness specifically, structural asymmetries in the motor cortex have been suggested to be of relevance (Amunts et al. 1996). A large-scale study of 106 left-handers and 1960 right-handers found a nominal significant association of left precentral sulcus surface area with left-handedness that did not, however, survive correction for multiple comparisons (Guadalupe et al. 2014).

Based on the previously described literature, the present study had the following hypotheses. First, we hypothesized that GWAS-derived PGS for handedness should be significantly associated with handedness phenotypes in our validation sample. Moreover, we explored to what extent handedness PGS are associated with asymmetries in gray matter brain structure. Here, we hypothesized that if significant associations are present, they should be found primarily for motor cortex areas and, therefore, also specifically assessed the precentral gyrus. The results of these analyses are also of interest in the context of the question whether or not different forms of lateralization are determined by the same or different underlying genetic factors.

## Methods

### Participants

Overall, 320 healthy adult participants (167 males and 153 females) took part in the present study. After quality control of genetic data (see below), the final sample consisted of 296 participants (155 males and 141 females). Mean age was 27.67 years (standard deviation 10.53, range 18–75 years). Participants had no history of neurological or psychiatric disorders according to self-report. We did not deliberately oversample for left-handedness. Thus, handedness distribution was population-based. The study was approved by the local ethics committee of the Faculty of Psychology at Ruhr University Bochum, Germany. All participants gave written informed consent and were treated in accordance with the Declaration of Helsinki.

### Handedness assessment

Handedness was assessed using the EHI (Oldfield 1971). Participants had to answer ten items regarding the hand they preferred to use for various activities like writing and drawing. Based on these answers, a lateralization quotient (LQ) was determined using the following formula: LQ = [(R−L)/(R + L)]×100. In this formula, “R” indicates the number of answers for the right hand and “L” indicates the number for the left hand. The LQ is a continuous variable with a range between −100 (consistent left-handedness) and 100 (consistent right-handedness), with 0 indicating ambilaterality. The LQ is a composite score reflecting both direction of handedness (negative values indicate a leftward preference and positive values indicate a rightward preference) and strength of handedness (values close to zero indicate low handedness strength and values close to 100/−100 indicate high handedness strength). Based on handedness LQ, we also determined two additional phenotypes. Handedness strength was defined as the absolute LQ independent of direction (e.g., a LQ value of 100 and a LQ value of −100 would both be a handedness strength value of 100). Handedness direction was based on the sign of the LQ. Individuals with negative LQ values were classified as left-handed and individuals with positive LQ values as right-handed.

### DNA sampling and genotyping

For non-invasive sampling, exfoliated cells were brushed from the oral mucosa of the participants. DNA isolation was performed with QIAamp DNA mini Kit (Qiagen GmbH, Hilden, Germany). Genotyping was carried out using the Illumina Infinium Global Screening Array 1.0 with MDD and Psych content (Illumina, San Diego, CA, USA) at the life and brain facilities, Bonn, Germany. Filtering was performed with PLINK 1.9 (Chang et al. 2015; Purcell et al. 2007) removing SNPs with a minor allele frequency of  < 0.01, deviating from Hardy–Weinberg equilibrium with a *p* value of  < 1×10^–6^, and missing data  > 0.02. Participants were excluded with  > 0.02 missingness, sex-mismatch, and heterozygosity rate >|0.2|. Filtering for relatedness and population structure was carried out on a SNP set filtered for high quality (HWE *p* > 0.02, MAF > 0.2, missingness = 0), and LD pruning (*r*^2^ = 0.1). In pairs of cryptically related subjects (pi hat  > 0.2), one subject was excluded at random. Principal components to control for population stratification were generated, and outliers >|6SD| on one of the first 20 PC were excluded. The final data set consisted of 296 participants and 491,138 SNPs.

### Polygenic score analysis

PGS for each participant were created using publicly available summary statistics for left-handedness, right-handedness, and ambidexterity based on the results of the three GWAS in the study by de Kovel and Francks (2019). PGS were calculated as the weighted sums of each participant’s trait-associated alleles across the SNPs retained after clumping (250 kb window, *r*^2^ > 0.1) with PRSice-2 software using standard settings (version 2.1.6) (Choi and O’Reilly 2019). The best-fit approach (Choi and O’Reilly 2019) was applied to empirically determine the *p* value threshold (PT) for inclusion of SNPs (for the range of *p* value threshold from 0.0001 to 0.5 in steps of 5×10^–5^). The respective best-fit PGS explained a maximum amount of variance in handedness LQ in our sample. The so-called ‘incremental *R*^2^’ statistic was used to determine the predictive power of the handedness PGS derived from the three GWAS. This statistic reflects the increase in the determination coefficient (*R*^2^) when the PGS is added to a regression model that predicts the handedness LQ and includes control variables (here sex, age, and the first four principal components of population stratification). In addition to the analyses with handedness LQ, we also conducted the same analyses for handedness strength and handedness direction. For all statistical analyses in PRSice-2, linear parametric methods were used. Testing was two-tailed with an α-level of *p* < 0.05. Subsequently, the best-fit PGS for left-handedness, right-handedness and ambilaterality were used to investigate associations with neuroimaging measures (see below). PGS and other data will be made available upon reasonable request.

### Neuroimaging

Anatomical neuroimaging data were acquired using a 3 T Philips Achieva MRI scanner outfitted with a 32-channel head coil. The MRI scanner was located at Bergmannsheil University Hospital in Bochum, Germany. MRI scans of each participant were acquired using a T1-weighted high-resolution anatomical imaging MP-RAGE sequence. The following parameters were used: repetition time = 8.2 ms, echo time = 3.7 ms, flip angle = 8°, 220 slices, matrix size = 240 × 240, resolution = 1 × 1×1 mm, acquisition time = 6 min. Reconstruction of cortical surface, volume and thickness within the T1-weighted images was performed using FreeSurfer software (http://surfer.nmr.mgh.harvard.edu, version 6.0.0), following previously established protocol (Dale et al. 1999; Fischl et al. 1999). Pre-processing of the MRI images was performed automatically for each participant and consisted of skull stripping and gray matter segmentation, followed by reconstruction and inflation of the cortical surface. Subsequently, manual quality control was performed slice by slice and potential inaccuracies of automatic preprocessing were corrected manually. Overall, 34 cortical brain regions were extracted for each hemisphere based on an established labeling system for subdividing the cortex in MRI scans into gyral based regions of interest (Desikan et al. 2006). This procedure was performed using an automatic segmentation procedure implemented in FreeSurfer. Based on the parameters obtained for the 34 cortical brain regions in the left and the right hemisphere, three different LQs were determined for each brain area (one for cortical surface, one for cortical volume, and one for cortical thickness).

To ensure comparability with the handedness data, the anatomical LQs were determined using the following formula: LQ = [(R−L)/(R + L)]×100. In this formula, “R” indicates the parameter (surface, volume or thickness) obtained for a specific brain structure in the right hemisphere. In contrast, “L” indicates the parameter (surface, volume or thickness) obtained for a specific brain structure in the left hemisphere. Thus, a positive LQ reflects a rightward structural asymmetry and a negative LQ a leftward structural asymmetry.

## Results

### Distribution of handedness LQ and PGS

**Fig. 1:**
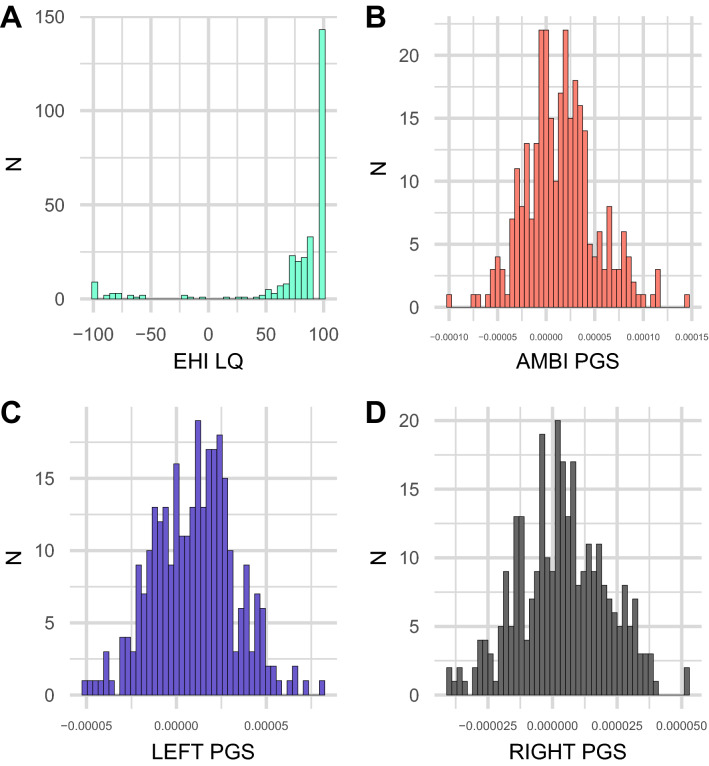
Distributions of individual values for **A** handedness LQ, **B** ambilaterality PGS, **C** left-handedness PGS, and **D** right-handedness PGS. *Y*-axis shows number of participants. *X*-axis shows LQ or PGS values

The distributions of handedness LQ and the three different PGS are shown in Fig. 1. The average handedness LQ was 74.23 (SD = 49.32) with a range of −100–100. It showed a typical J shaped distribution with a strong skew to the right end of the distribution, reflecting that most participants were right-handed. Overall, 26 participants (8.8%) had an LQ below zero, indicating left-handedness and 270 participants (91.2%) had an LQ above zero, indicating right-handedness. All three PGS were normally distributed (Kolmogorov–Smirnov test for normal distribution; right-handedness PGS *p* = 0.99, left-handedness PGS *p* = 0.92, ambilaterality PGS *p* = 0.29), while LQ was not normally distributed (*p* < 0.001).

### Association of PGS and handedness LQ

**Fig. 2:**
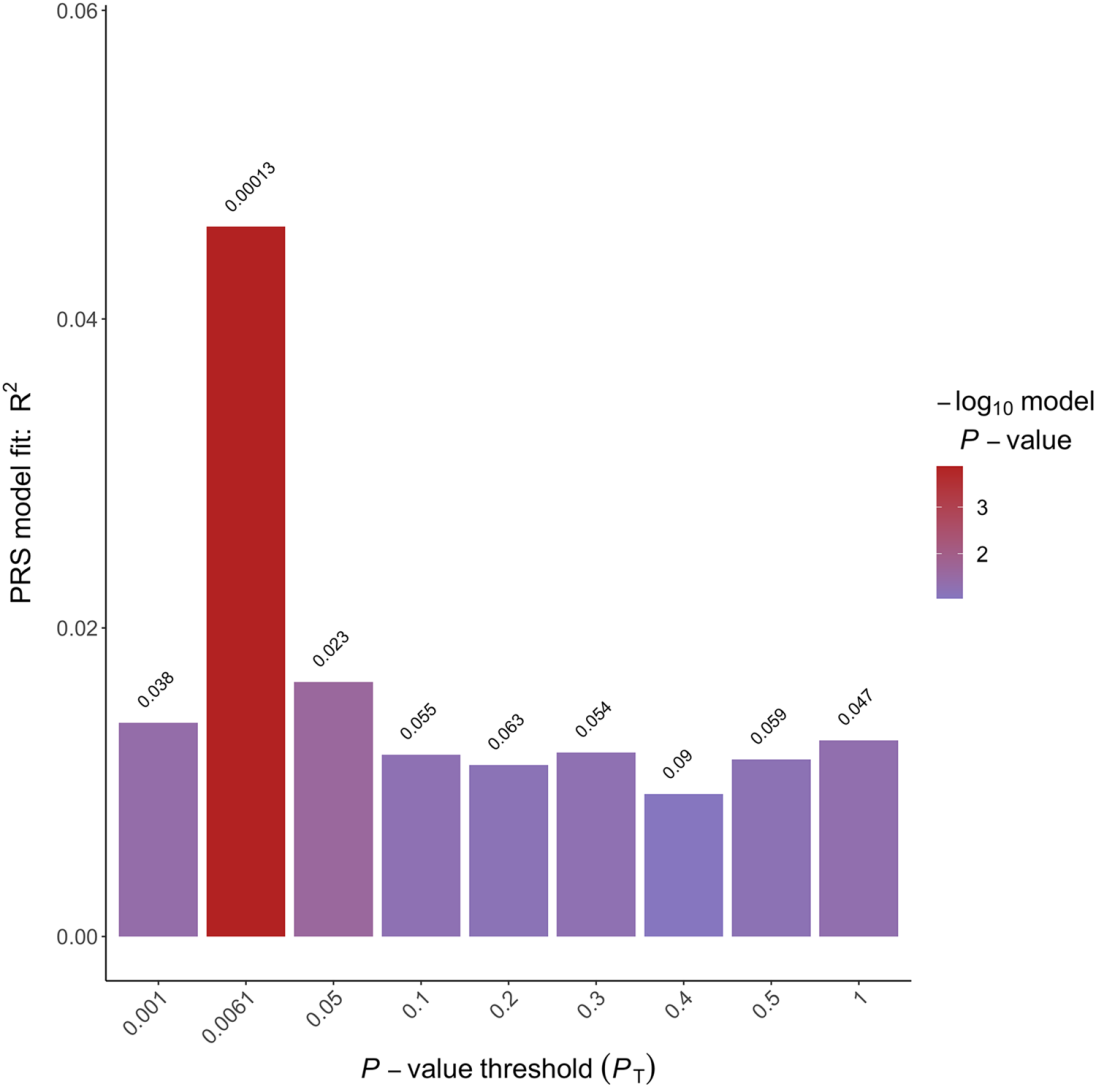
Incremental *R*^2^ of the best-fit polygenic scores of right-handedness PGS in percent. The *p* value thresholds that determined the inclusion of SNPs into the respective PGS are displayed over each bar. The incremental *R*^2^ reflects the increase in the determination coefficient (*R*^2^) when the PGS are added to a regression model predicting individual differences in handedness LQ. The association between PGS and phenotype was controlled for the effects of sex, age, and population stratification

**Fig. 3:**
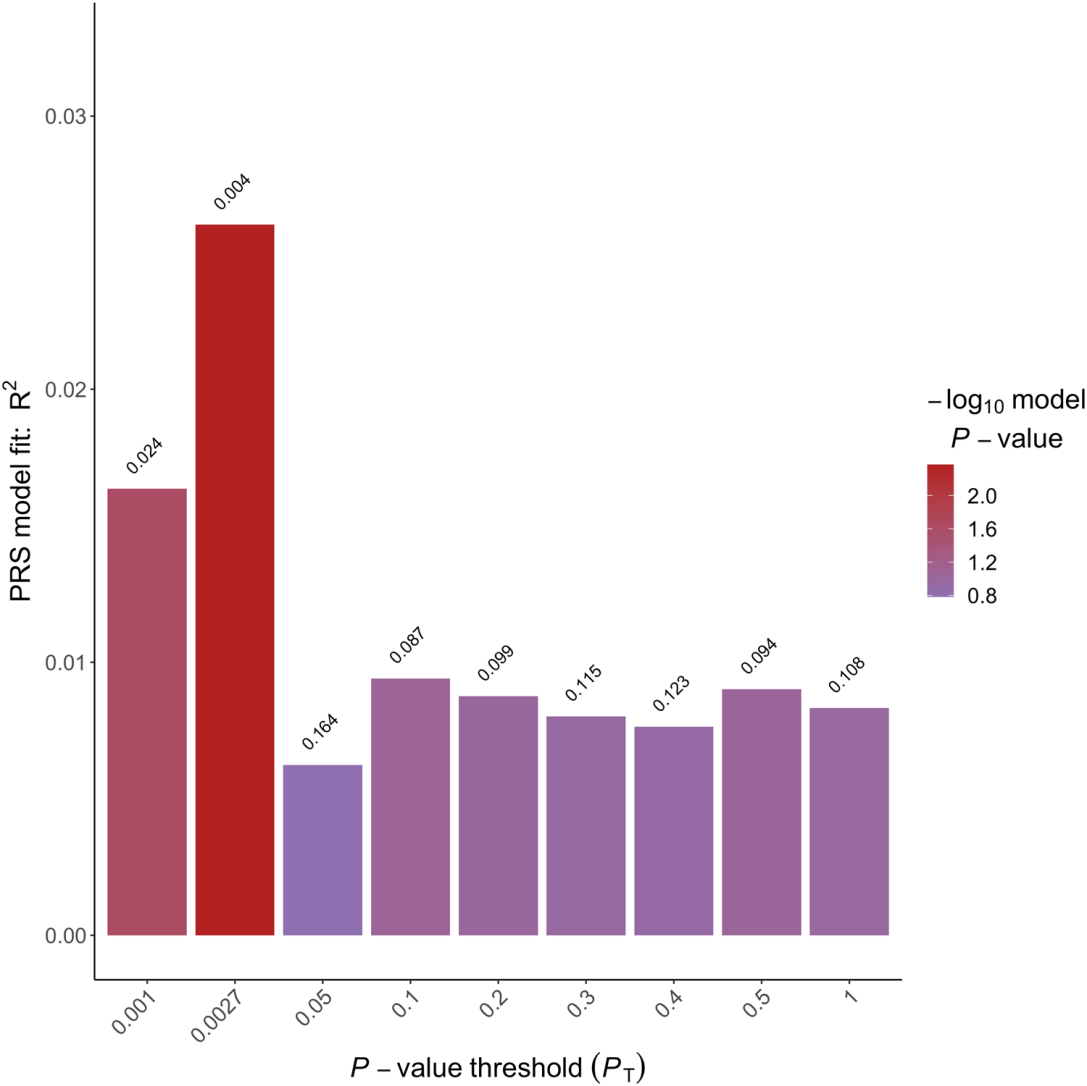
Incremental *R*^2^ of the best-fit polygenic scores of left-handedness PGS in percent. The *p* value thresholds that determined the inclusion of SNPs into the respective PGS are displayed over each bar. The incremental *R*^2^ reflects the increase in the determination coefficient (*R*^2^) when the PGS are added to a regression model predicting individual differences in handedness LQ. The association between PGS and phenotype was controlled for the effects of sex, age, and population stratification

**Fig. 4:**
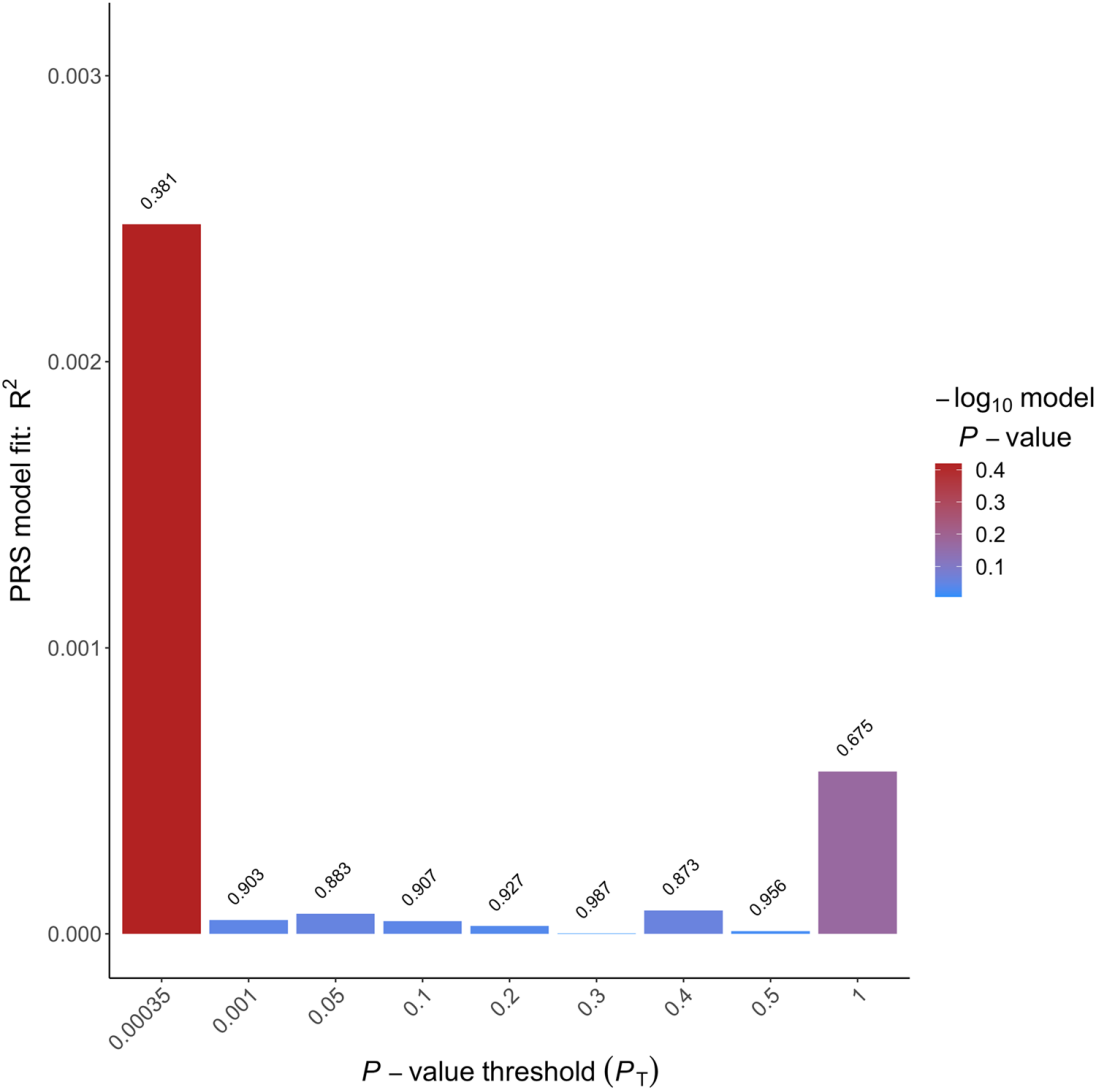
Incremental *R*^2^ of the best-fit polygenic scores of ambilaterality PGS in percent. The *p* value thresholds that determined the inclusion of SNPs into the respective PGS are displayed over each bar. The incremental *R*^2^ reflects the increase in the determination coefficient (*R*^2^) when the PGS are added to a regression model predicting individual differences in handedness LQ. The association between PGS and phenotype was controlled for the effects of sex, age, and population stratification

Right-handedness PGS (see Fig. 2) were significantly associated with individual LQ [*P*_T_ = 0.0061, incremental *R*^2^ = 4.60% (95% CI (1.32, 11.53%), *p* = 0.00013]. Higher right-handedness PGS were associated with a higher positive LQ indicating stronger right-handedness. Similarly, left-handedness PGS (see Fig. 3) were significantly associated with individual LQ [*P*_T_ = 0.0027, incremental *R*^2^ = 2.60% (95% CI (1.11, 11.19%), *p* = 0.004]. Here, higher left-handedness PGS were associated with a higher negative LQ indicating stronger left-handedness. In contrast, ambilaterality PGS (see Fig. 4) were not significantly associated with individual LQ (*p* = 0.381). As handedness LQ was not normally distributed, we confirmed these results for the respective best-fit *P*_T_ using non-parametric testing. We determined non-parametric partial correlation coefficients (Spearman’s *ρ*, two-tailed testing) between handedness LQ and the three PGS with the same control variables as in the parametric analyses (sex, age, and the first four principal components of population stratification). Results were similar to the parametric analysis. Right-handedness PGS were significantly correlated with individual LQ (*ρ* = 0.23, *p* = 0.000084), as was left-handedness PGS (*ρ* = −0.18, *p* = 0.0026). In contrast, ambilaterality PGS did not show a significant correlation with handedness LQ (*ρ* = 0.02, *p* = 0.29).

In addition to handedness LQ, we also determined the associations of PGS for handedness strength (Supplementary Figures S1–S3) and handedness direction (Supplementary Figures S4–S6) with the respective phenotypes in our cohort. For handedness strength, right-handedness PGS (see Figure S1) were significantly associated with individual handedness strength (*p* = 0.002), as were left-handedness PGS (see Figure S2, *p* = 0.021), and ambilaterality PGS (see Figure S3, *p* = 0.032). For handedness direction, right-handedness PGS (see Figure S4) were significantly associated with individual handedness direction (*p* = 0.00042), as was left-handedness PGS (see Figure S5, *p* = 0.009), but not ambilaterality PGS (see Figure S6).

### Correlations between PGS

At the respective best-fit *P*_T_ values, left-handedness PGS showed a significant negative correlation with right-handedness PGS (*r* = −0.62, *p* < 0.000001), but no correlation with ambilaterality PGS (*r* = 0.03, *p* = 0.96). Right-handedness PGS also did not show correlation with ambilaterality PGS (*r* = −0.07, *p* = 0.26).

### Correlations between PGS and brain structure LQs

**Fig. 5:**
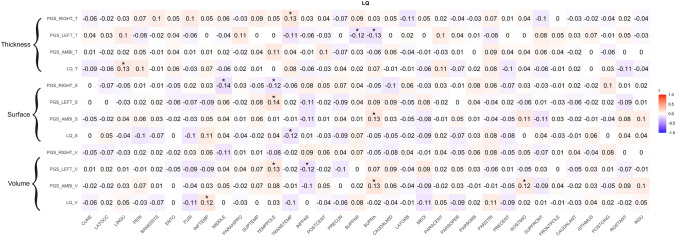
Pearson correlation coefficients between gray matter structure LQs (cortical thickness, surface, volume) and right-handedness PGS, left-handedness PGS, ambilaterality PGS as well as handedness LQ for the whole sample. ^*^p < 0.05, ^**^p < 0.01, ^***^p < 0.001. Bonferroni-corrected significance threshold is *p* = 0.00147

**Fig. 6:**
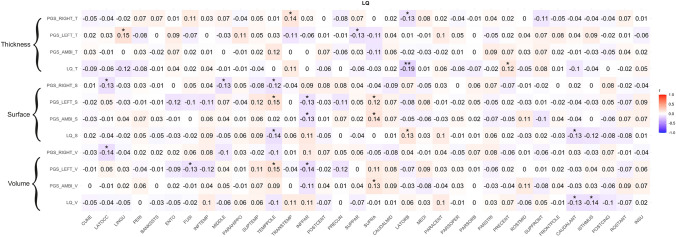
Pearson correlation coefficients between gray matter structure LQs (cortical thickness, surface, volume) and right-handedness PGS, left-handedness PGS, ambilaterality PGS as well as handedness LQ for right-handers only. ^*^p < 0.05, ^**^p < 0.01, ^***^p < 0.001. Bonferroni-corrected significance threshold is *p* = 0.00147

To investigate the relation of PGS and handedness LQ to asymmetries in gray matter structure, we correlated the three PGS and handedness LQ with LQs for cortical volume, surface, and thickness of the 34 brain regions using partial correlation coefficients including the control variables sex, age, and the first four principal components of population stratification. The threshold for nominal significance was set to *p* = 0.05. Since 34 different brain areas were investigated, the Bonferroni-corrected significance threshold was set to 0.05/34 = 0.00147. This was done for the whole sample (*n* = 296) (see Fig. 5), as well as only for right-handers (*n* = 270) (see Fig. 6). We do not report the findings for only left-handers, due to the small sample size of this group (*n* = 26). For the whole sample, none of the comparisons reached significance after correction for multiple comparisons (range of *r* between −0.14 and 0.14). For the subsample of right-handers, also none of the correlations reached significance after correction for multiple comparisons (range of *r* −0.19–0.15).

### Association between precentral gyrus LQ and handedness

Since previous studies reported specific associations between handedness and the precentral gyrus, we investigated structure–function relationships for this brain area in more detail. Using independent sample *t* test, we compared precentral gyrus LQs for volume, surface, and thickness between left-handers and right-handers. For volume LQ, there was a nominally significant effect (*t*_(294)_ = 2.38, *p* = 0.018), indicating that left-handers (1.21, SD = 3.58) had a more positive LQ than right-handers (−0.49, SD = 3.48). This indicates a rightward asymmetry in left-handers, but a leftward asymmetry in right-handers. For thickness LQ, there also was a nominally significant effect (*t*_(294)_ = 2.60, *p* = 0.0097), indicating that left-handers (0.11, SD = 1.30) had a more positive LQ than right-handers (−0.53, SD = 1.29). This indicates a rightward asymmetry in left-handers, but a leftward asymmetry in right-handers. For surface LQ, the effect failed to reach significance (*p* = 0.08). However, none of these effects came close to the significance threshold after correction for multiple comparisons (*p* = 0.00147).

## Discussion

Handedness is the most widely investigated form of motor preferences in humans (Güntürkün et al. 2020; Papadatou–Pastou et al. 2020; Paracchini et al. 2016), but both its relation to brain structure and the role of its genetic determinants for this relation are still largely unclear. Recent large-scale GWAS (Cuellar–Partida et al. 2020; de Kovel and Francks 2019; Wiberg et al. 2019) have advanced our understanding of the genetic factors involved in handedness ontogenesis, but the transfer of these insights into smaller-scale studies has not been explored yet. PGS have been suggested to substantially increase predictive power over single gene candidate studies (Dima and Breen 2015), while simultaneously also generating more replicable results than candidate gene studies, as PGS are based on the summary statistics of large, well-powered GWAS.

It was the aim of the present study to establish the use of PGS in handedness research in a sample of 296 healthy adults. We determined PGS for left-handedness, right-handedness, and ambilaterality based on the summary statistics of a recent GWAS by de Kovel and Francks (2019). PGS for left-handedness and right-handedness significantly were significantly associated with individual LQ with an incremental *R*^2^ of 4.6% for the right-handedness PGS and an incremental *R*^2^ of 2.6% for the left-handedness PGS. The ambilaterality PGS failed to reach significance, but this is no surprise considering the GWAS for ambidextrous vs. non-ambidextrous in the paper by de Kovel and Francks (2019) showed no significant associations. This was potentially due to the fact that the ambidextrous group was substantially smaller than the two other groups (*n* = 5324, compared to left-handers *n* = 31,856 and right-handers *n* = 293,857).

The incremental *R*^2^ statistics are within the range that can be expected based on the limited relevant literature. For handedness, the amount of phenotypic variance explained by non-genetic factors generally is larger than the amount of variance explained by genetic factors. For example, a study using a twin design found that additive genetic effects accounted for 25.47% of the phenotypic variance for handedness (Medland et al. 2006). Moreover, a recent study estimated SNP-based heritability for handedness to be somewhere between 3 and 6% (Cuellar–Partida et al. 2020). It has to be noted, that the best-fit approach we chose to select the *p* value thresholds for the subsequent imaging analysis potentially leads to an overestimation of the association of the PGS with handedness LQ. However, the effect direction was consistent over all of the predefined thresholds depicted in Figs. 2–4.

This was the first study to explore the use of PGS in handedness research. Our results implicate that PGS obtained from large handedness GWAS with simple phenotyping like the work of de Kovel and Francks (2019) show significant associations with handedness phenotypes in smaller samples like the present one. Moreover, an important insight was that the PGS that were determined based on a GWAS with categorical data (participants were classified as left-handers vs non-left-handers for the left-handedness GWAS, as right-handers vs non-right-handers for the right-handedness GWAS, and ambilateral vs non-ambilateral individuals for the ambilaterality GWAS) are associated with the LQ, an interval-scaled measure of handedness. While the LQ gives more information on individual handedness than just the distinction between left-handedness and right-handedness, large-scale GWAS typically have light phenotyping and might not include the EHI. Our results implicate that the summary statistics from such studies can still be used to generate PGS that show significant association with the LQ in validation samples.

Many of the top hit SNPs observed in the GWAS by de Kovel and Francks (2019) and other GWAS on handedness (Cuellar–Partida et al. 2020; Wiberg et al. 2019) are functionally involved in neurogenesis and early brain development such as *MAP2* (Harada et al. 2002). Therefore, we assessed the association of handedness PGS and asymmetries in gray matter volume, thickness, and surface area. These brain phenotypes might be associated with handedness, specifically for motor areas (Amunts et al. 1996, 2000; Guadalupe et al. 2014). This was done as PGS on their own have very limited potential to allow for any functional insights into how the genetic variation reflected by them shapes a complex phenotype like human motor behavior. Exploring their association with brain phenotypes could be informative for understanding the link between genetic variation and behavioral phenotypes on a functional level. However, the results of the present study suggest that at least for gray matter volume, thickness, and surface area in specific brain areas, the predictive power of handedness-based PGS in the overall sample was low. The range of *r* values in the overall sample was between −0.14 and 0.14 and in fact, none of the correlations between PGS and measures of structural asymmetries reached significance after correction for multiple comparisons. The fact that we did not find any relation between handedness PGS and gray matter structural asymmetries in the overall sample might be attributed to a weak relationship between handedness and macrostructural gray matter asymmetries. Significant differences between left and right-handers regarding structural asymmetries in motor areas have been reported previously (Amunts et al. 1996). However, in a large-scale study, no difference between left and right-handers survived correction for multiple comparisons (Guadalupe et al. 2014). In this study, a nominal significant association of left precentral sulcus surface area with left-handedness was observed. Due to this result, we specifically investigated the relation of the structural LQs for the precentral gyrus (the precentral sulcus was not included in the parcellation scheme used in the present study). Somewhat in line with the findings of Guadalupe, we also observed two nominal significant effects (for volume LQ and thickness LQ) that failed to reach significance after correction for multiple comparisons. This suggests that the relationship between handedness and gray matter asymmetries of the precentral gyrus as defined in the parcellation scheme by Desikan et al. (2006) is weak. However, given our results and those of previous studies, some associations between handedness and gray matter asymmetries in motor areas seem to exist. One potential explanation for this result could be that handedness is associated with structural asymmetries of specific hand representation areas in the precentral gyrus (Hanakawa et al. 2005), but not with structural asymmetries in the whole precentral gyrus. Using high-resolution imaging to determine specific areas involved in the neural representation of fingers (Yokoi et al. 2018) and relating structural asymmetries in these areas to handedness may be a meaningful way to empirically test this assumption. Moreover, layer-specific fMRI might also be helpful in understanding specifically which cortical layers are relevant for handedness (Persichetti et al. 2020), as recent primate research suggests the existence of layer-specific structural asymmetries in the primate brain (Contestabile et al. 2020). In general, it would be highly useful to use fMRI to determine whether functional hemispheric asymmetries in brain activation may be a phenotype that mediates between genetic variation reflected by PGS and behavioral laterality phenotypes like handedness. For example, individual activation asymmetries during motor tasks that have been shown to generate significant differences between left and right-handers (Klöppel et al. 2007) may correlate with handedness PGS to a greater extent than structural asymmetries.

While our study provided first insights into the use of PGS in laterality research, several methodological considerations must be taken into account when interpreting the present results. First, the low number of left-handers might have limited insights in relation to handedness as a categorical phenotype. Future studies should establish the use of handedness PGS to predict left-handedness vs right-handedness as a categorical variable in samples that contain a larger number of left-handed individuals.

Second, our sample is comparatively small for a genetic study with less than 300 participants. The *R*^2^ estimated with the best-fit approach may potentially be an overestimation of the real effect due to the small sample sizes. Related to this issue is also a relatively strong skewedness of the phenotypic data (the typical J shaped distribution of the LQ data). Replication in larger cohorts is, therefore, necessary.

Third, our study was limited to assessing handedness in the form of hand preference and strength as assessed with the EHI. However, some previous studies on the genetics of handedness (Scerri et al. 2011) have also used measures of hand skill such as the peg board task. Thus, future studies on handedness PGS should use both measures of hand preference and hand skill.

Fourth, studies on hemispheric asymmetries could also utilize other forms of PGS than those obtained from handedness GWAS. For example, exploring the predictive power of PGS obtained for clinical diagnoses such as schizophrenia for functional and structural hemispheric asymmetries could be helpful for getting a better understanding of the core question of clinical laterality research: Why are so many neurodevelopmental and psychiatric disorders associated with atypical hemispheric asymmetries (Mundorf and Ocklenburg 2021)? One study (Whalley et al. 2015) used PGS for schizophrenia to predict brain activation while participants performed a language-based executive task and found a specific effect for left lateral frontal brain activation. Future studies should use genomic approaches to study the link between disorders associated with atypical hemispheric asymmetries such as autism spectrum disorders (Lindell and Hudry 2013), dyslexia (Brandler and Paracchini 2014), and PTSD (Zach et al. 2016) and asymmetry phenotypes. Importantly, a recent study on the genetic architecture of structural hemispheric asymmetries in the human brain suggested that genetic variants affecting brain asymmetry overlapped with those influencing autism and schizophrenia, but also education attainment (Sha et al. 2021).

In conclusion, the present study is the first to investigate associations between GWAS-derived PGS and handedness LQ, a continuous phenotype. It was shown that handedness PGS are associated with phenotypic variation in a validation sample much smaller than the GWAS they were based on. Moreover, the results suggest that different genetic factors are relevant for asymmetries in gray matter structure than for handedness.

## Supplementary Information

Below is the link to the electronic supplementary material.

Supplementary file1 (DOCX 744 KB)

## Data Availability

Data will be made available upon reasonable request.

## Code availability

Code will be made available upon reasonable request.
