# Supplementary material for: Polygenic scores for handedness and their association with asymmetries in brain structure
Source: Brain Struct Funct. 2021 Jul 8;227(2):515–27. doi: 10.1007/s00429-021-02335-3 (PMC8844179; doi:10.1007/s00429-021-02335-3)
Supplement: Supplementary file 1 — Supplementary file1 (DOCX 744 KB) [file 429_2021_2335_MOESM1_ESM.docx]

**Supplementary Materials**

**
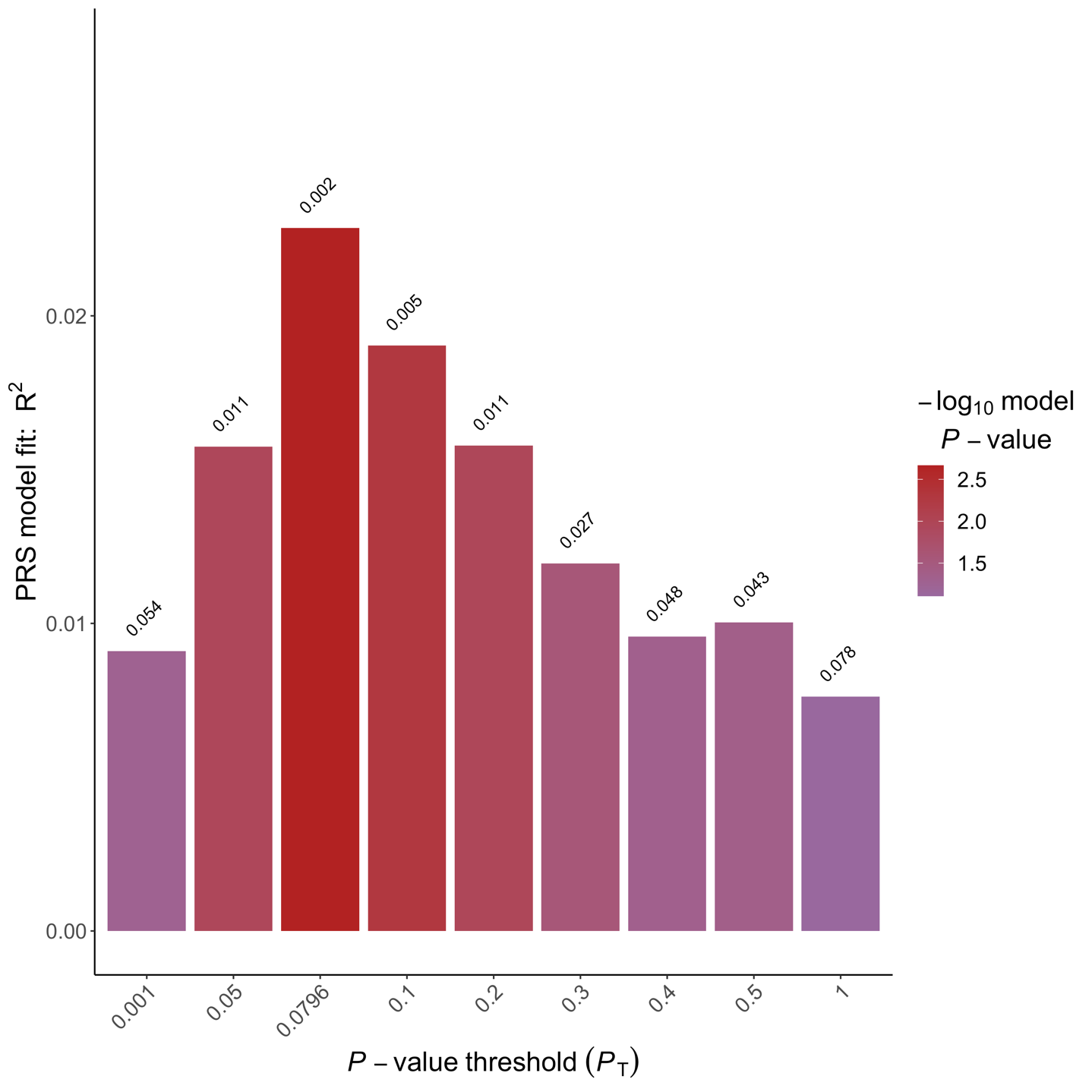
**

**Figure S1**: PGS for right-handedness in relation to handedness strength. Incremental R^2^ of the best-fit polygenic scores of right-handedness PGS in percent. The p-value thresholds that determined the inclusion of SNPs into the respective PGS are displayed over each bar. The incremental R^2^ reflects the increase in the determination coefficient (R^2^) when the PGS is added to a regression model predicting individual differences in handedness LQ strength. The association between PGS and phenotype was controlled for the effects of sex, age, and population stratification.


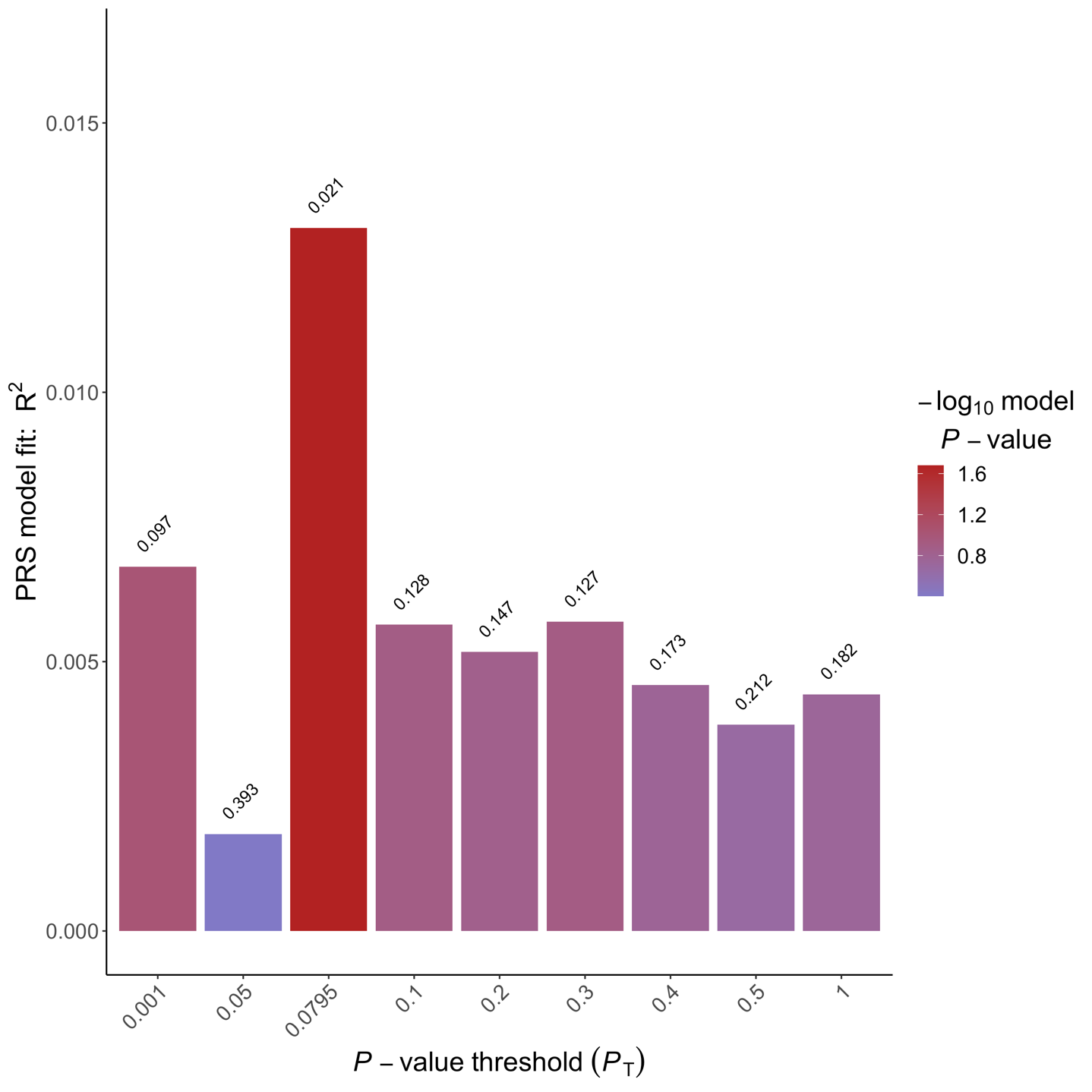


**Figure S2**: PGS for left-handedness in relation to handedness strength. Incremental R^2^ of the best-fit polygenic scores of left-handedness PGS in percent. The p-value thresholds that determined the inclusion of SNPs into the respective PGS are displayed over each bar. The incremental R^2^ reflects the increase in the determination coefficient (R^2^) when the PGS is added to a regression model predicting individual differences in handedness LQ strength. The association between PGS and phenotype was controlled for the effects of sex, age, and population stratification.

**
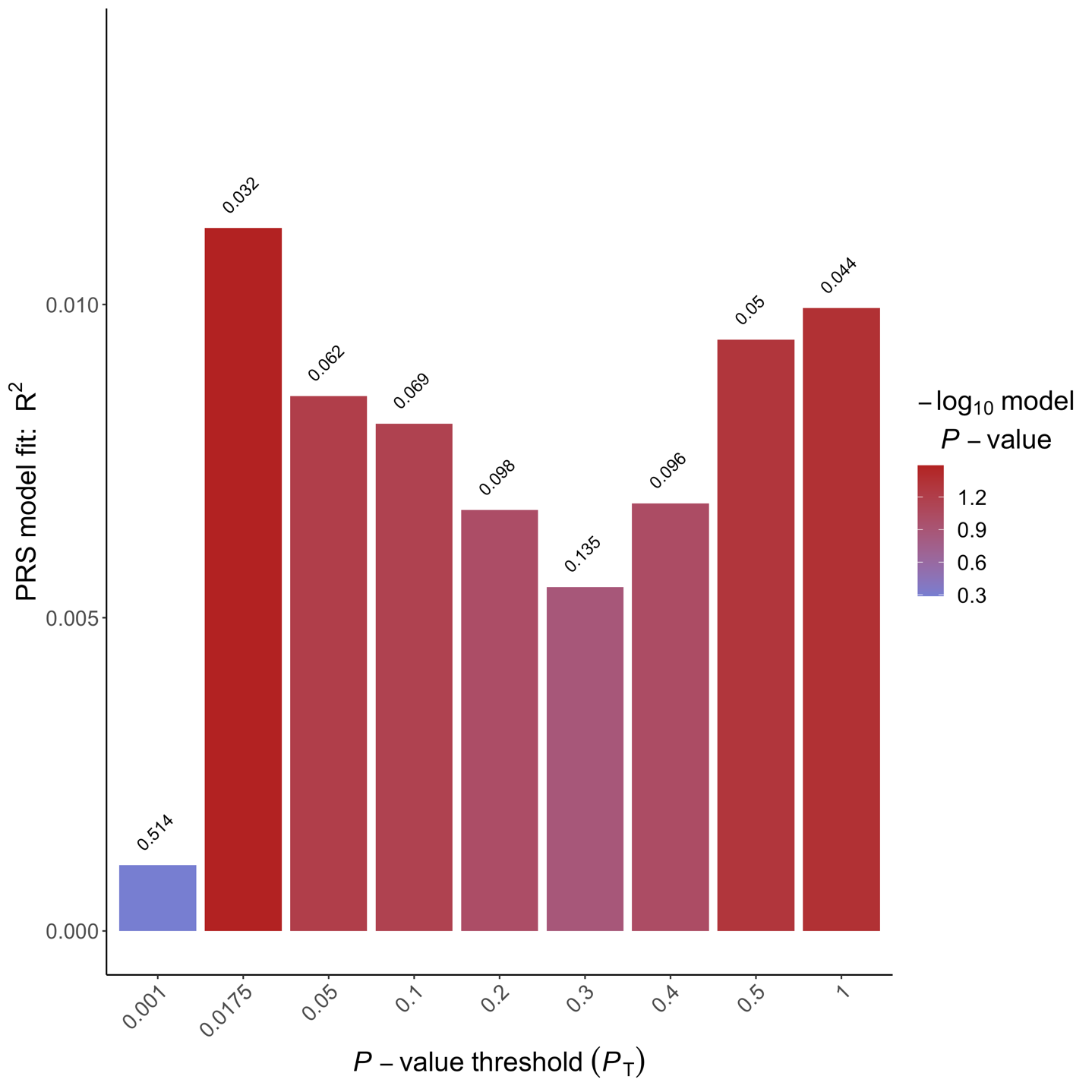
**

**Figure S3**: PGS for ambilaterality in relation to handedness strength. Incremental R^2^ of the best-fit polygenic scores of ambilaterality PGS in percent. The p-value thresholds that determined the inclusion of SNPs into the respective PGS are displayed over each bar. The incremental R^2^ reflects the increase in the determination coefficient (R^2^) when the PGS is added to a regression model predicting individual differences in handedness LQ strength. The association between PGS and phenotype was controlled for the effects of sex, age, and population stratification.

**
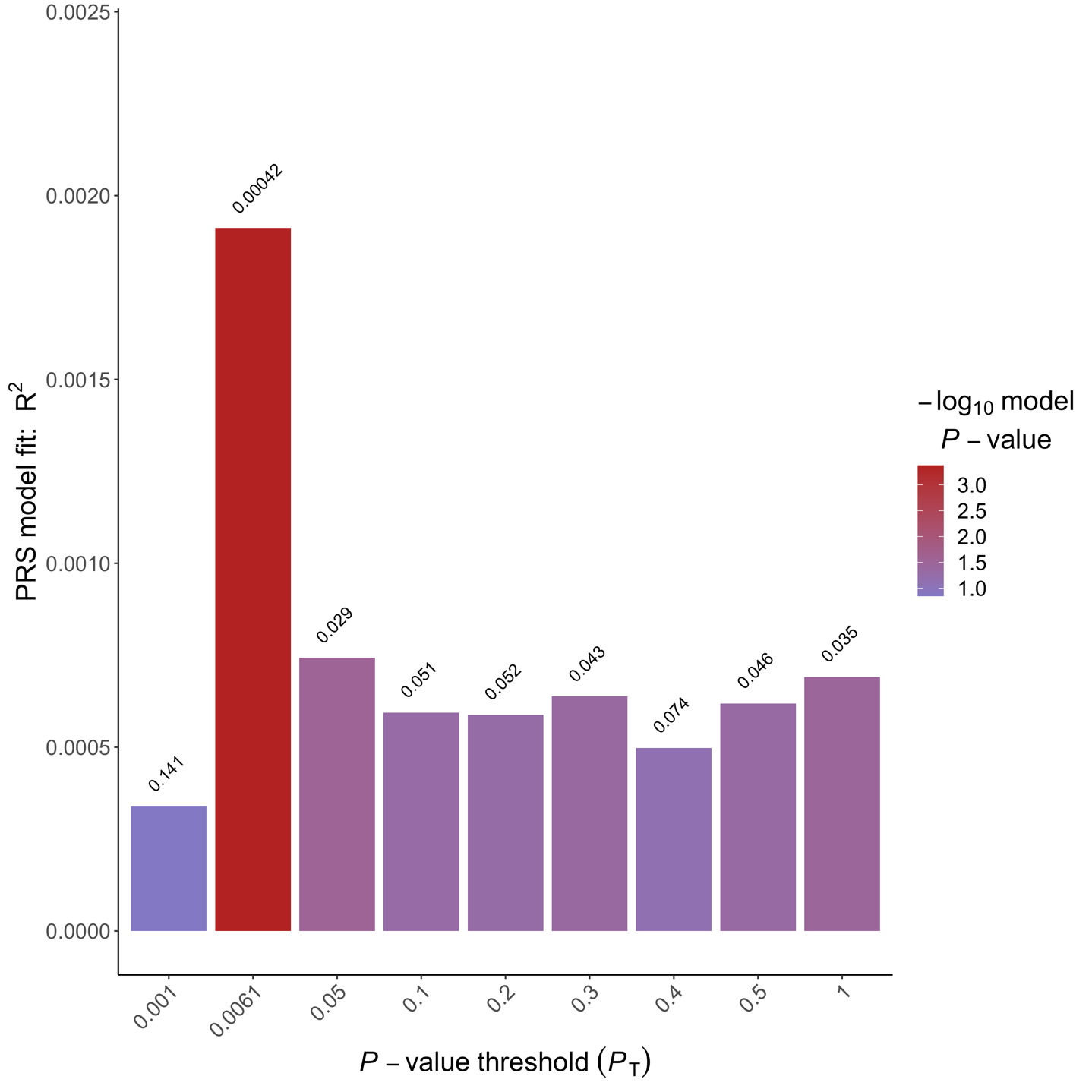
**

**Figure S4**: PGS for right-handedness in relation to handedness direction. Incremental R^2^ of the best-fit polygenic scores of right-handedness PGS in percent. The p-value thresholds that determined the inclusion of SNPs into the respective PGS are displayed over each bar. The incremental R^2^ reflects the increase in the determination coefficient (R^2^) when the PGS is added to a regression model predicting individual differences in handedness LQ direction. The association between PGS and phenotype was controlled for the effects of sex, age, and population stratification.

**
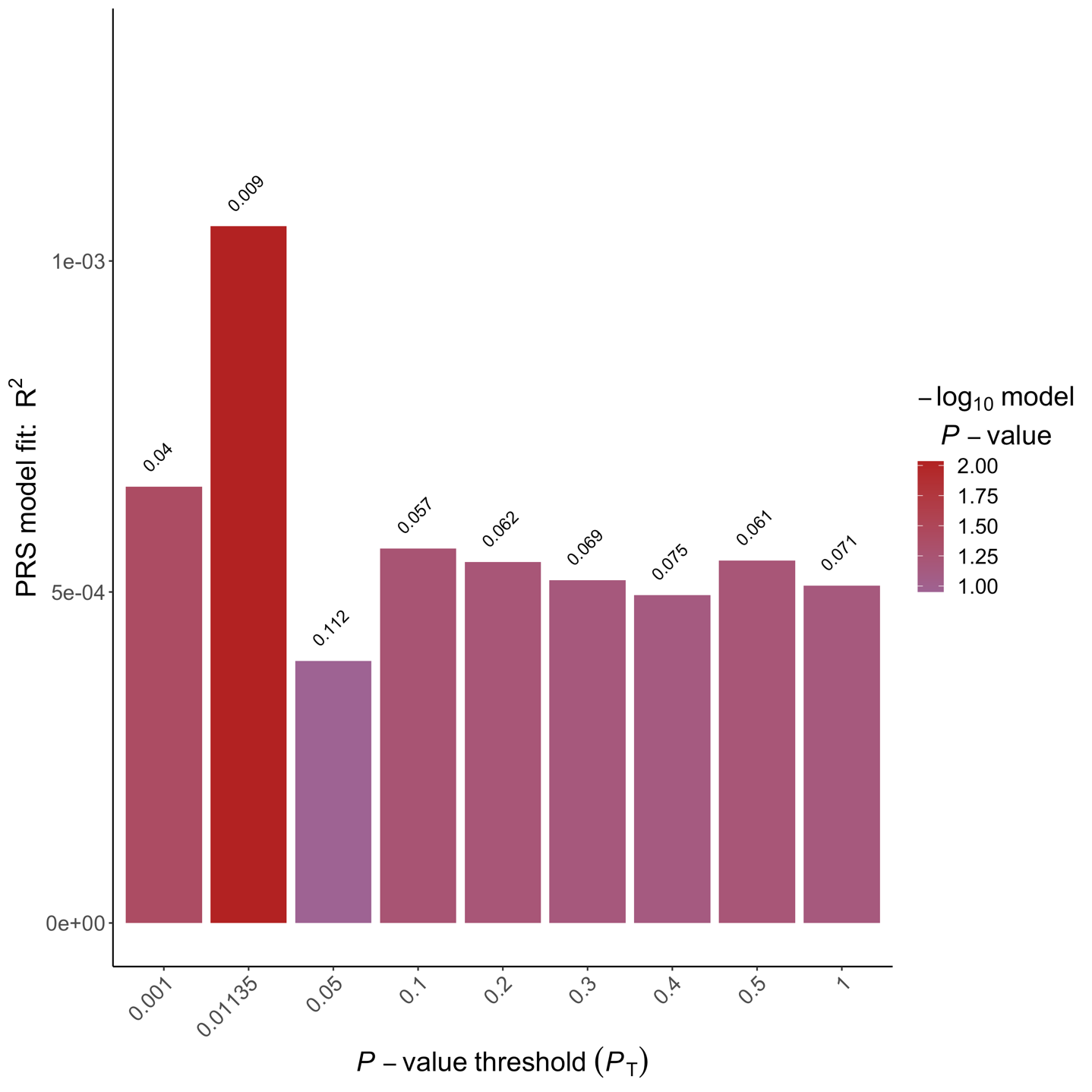
**

**Figure S5**: PGS for left-handedness in relation to handedness direction. Incremental R^2^ of the best-fit polygenic scores of left-handedness PGS in percent. The p-value thresholds that determined the inclusion of SNPs into the respective PGS are displayed over each bar. The incremental R^2^ reflects the increase in the determination coefficient (R^2^) when the PGS is added to a regression model predicting individual differences in handedness LQ direction. The association between PGS and phenotype was controlled for the effects of sex, age, and population stratification.

**
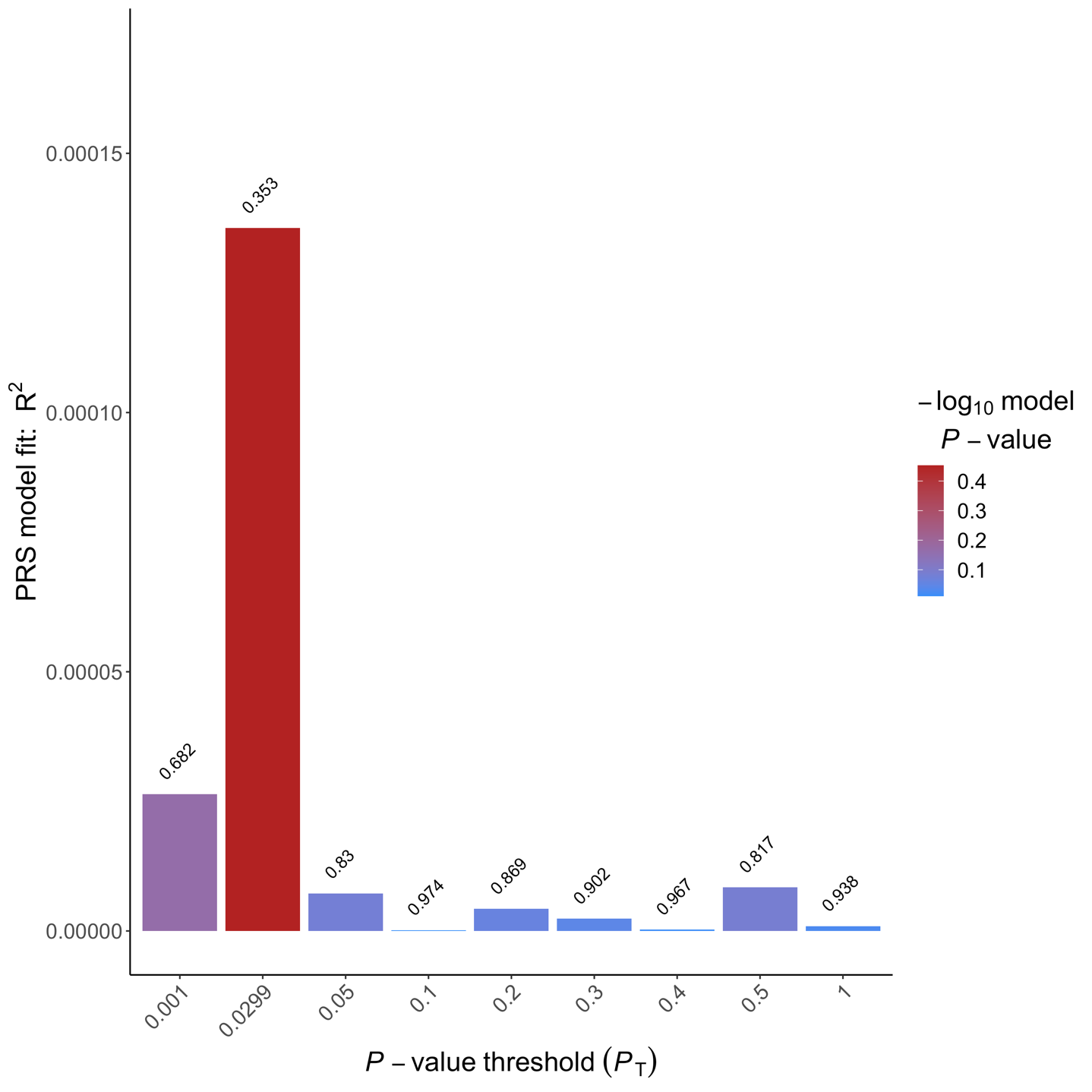
**

**Figure S6**: PGS for ambilaterality in relation to handedness direction. Incremental R^2^ of the best-fit polygenic scores of ambilaterality PGS in percent. The p-value thresholds that determined the inclusion of SNPs into the respective PGS are displayed over each bar. The incremental R^2^ reflects the increase in the determination coefficient (R^2^) when the PGS is added to a regression model predicting individual differences in handedness LQ direction. The association between PGS and phenotype was controlled for the effects of sex, age, and population stratification.
